# Gut microbiome-induced metabolites promote the role of Silybin as adjunctive drug in HIV-positive immunological nonresponders

**DOI:** 10.1080/29933935.2025.2569789

**Published:** 2025-10-23

**Authors:** Wenli Liu, Minghui An, Qi Wang, Yun Liu, Yuxin Shang, Xue Dong, Haibo Ding, Shuai Fu, Xiaoxu Han, Hong Shang

**Affiliations:** a State Key Laboratory for Diagnosis and Treatment of Infectious Diseases, NHC Key Laboratory of AIDS Prevention and Treatment, National Clinical Research Center for Laboratory Medicine, The First Hospital of China Medical University, China Medical University, Shenyang, China; b Key Laboratory of AIDS Immunology, Chinese Academy of Medical Sciences, Shenyang, China; c Department of Laboratory Medicine, National Clinical Research Center for Laboratory Medicine, The First Hospital of China Medical University, Shenyang, China

**Keywords:** HIV-1, immunological nonresponders, silybin, gut microbiome, inflammation

## Abstract

HIV-infected immunological nonresponders (INRs) endure persistent T-cell dysfunction and chronic inflammation, facing high risk of various complications and mortality, with no effective therapies available. Silybin, the principal constituent of a plant extract, possesses anti-inflammation and immunomodulatory properties. The gut microbiome has been shown to modulate the efficacy of immune therapies and drugs. We gave 54 INRs oral silybin for three months and used multi-omics to investigate the gut-related factors influencing the efficacy of silybin. Silybin raised CD4^+^ T cells counts in 52% of participants and an efficacy classification model based on baseline gut microbiome and metabolites was developed. Favorable gut bacteria produced anti-inflammatory metabolites that downregulated Ras/MAPK/PI3K-Akt signaling pathways also targeted by silybin. Our findings shed light on a novel therapeutic approach for addressing immune dysfunction in HIV-positive INRs and have important implications for personalized medical strategies in the management of HIV infection.

## Introduction

Human immunodeficiency virus type 1 (HIV-1) infection is a severe global health issue, and by the end of 2024, approximately 40.8 million individuals were estimated to be infected with HIV worldwide by UNAIDS. Sustained loss of CD4^+^ T cells is an outstanding characteristic of HIV infection. Although successful antiretroviral therapy (ART) can achieve complete virological suppression, 10−40% of patients fail to recover their CD4^+^ T-cell counts under ART.^1^ They are defined as immunological nonresponders (INRs), with a higher risk of AIDS- or non-AIDS-related complications and mortality.

Preclinical and clinical studies have shown that INRs exhibit significant immune dysfunction with specific mechanisms, including decreased thymic activity, increased cellular depletion, residual viral replication, and abnormal immune activation.^2-4^ Compared to other HIV-1-infected patients who achieve complete immune reconstitution, INRs have a distinct distribution of T-cell subsets, increased activated/exhausted T cells, and obvious inflammation-related protein (IRP) characteristics^5^, resulting in a worse prognosis. Although some immune adjunctive drugs that can reduce activation and inflammation have been used to modulate the immune dysfunction of INRs, there are no effective measures to promote the recovery of CD4^+^ T cells in INRs.^6^ However, in a recent phase II clinical study including 149 INR patients, *Tripterygium wilfordii* Hook F was proven to promote CD4^+^ T cells recovery and decrease inflammation by inhibiting the interferon signaling pathway and phosphorylation of STAT1.^7^^,^^8^ Therefore, traditional plant drugs may play key roles in modulating immunity for INRs, which provides an innovative therapeutic concept.

Silymarin (SM) is an extract from the plant milk thistle and its main constituent silybin (SIL) accounts for approximately 60%−70% of silymarin and is widely utilized for the treatment of various liver diseases.^9^ Notably, SIL is the only liver-protecting plant-derived Chinese herbal medicine approved by the China National Medical Products Administration (NMPA). Additionally, SM plays important roles in anti-inflammation, antioxidation, antiapoptosis, and antiproliferation by multiple cellular and molecular pathways.^10^ During the SARS-CoV-2 pandemic, SIL was found to have anti-inflammatory and anticoagulant properties, along with its ability to interact with the primary SARS-CoV-2 target protein, making it a promising candidate for COVID-19 treatment.^11^ For HIV infection, both in vitro and mouse studies have shown that SM or SIL can inhibit the activation and proliferation of T cells, modify T-cell subsets, regulate Treg, Th1, and Th17, as well as reduce inflammatory cytokines.^12-14^ However, there is no direct clinical evidence that SIL can restore CD4^+^ T-cell counts and rectify immune dysfunction in INRs.

It is well known that the gut microbiome can exert immunoregulatory activity in health and various diseases directly or via their metabolites^15^^,^^16^ and can also contribute to the efficacy of immune therapies and drugs. Immune checkpoint inhibitors that target cytotoxic T lymphocyte-associated antigen (CTLA-4) and programmed death 1 (PD-1) proteins have made significant advancements in the treatment of melanoma and other cancers. However, the efficacy of these immune checkpoint inhibitors is often heterogeneous.^17-20^ Clinical and mouse studies have revealed that melanoma patients with a “favorable microbiome” respond well to anti-PD-1 immunotherapy via increased antigen presentation and improved effector T-cell function mediated by gut bacteria.^20^ Moreover, some mechanisms by which the microbiome enhances antitumor immunity, such as the bacteria-metabolite-immune pathway activated by immune checkpoint inhibitors, have been discovered.^21^ Similarly, the gut microbiome and its genes also affect drug efficacy through metabolism and chemical modification.^22^ The cardiac drug digoxin can be converted into inactivated dihydrodigoxin by the cardiac glycoside reductase encoded by *Eggerthella lenta.*^23^ The antidiabetic drug acarbose can be selectively phosphorylated by gut bacteria-derived acarbose kinases.^24^ Therefore, we believe that the combined gut microbiome may further modify the response to immune adjunctive drugs in the absence of effective measures for CD4^+^ T-cell recovery in INRs.

In this study, we investigated the effectiveness and heterogeneity of SIL as an immunotherapy in 54 HIV-positive INRs and explored how the gut microbiome at treatment initiation regulates and improves SIL effects. We found favorable bacteria and metabolites that contributed to a good response to SIL and inferred that the multi-regulation of SIL pathways induced by the microbiome–metabolite–cytokine network may promote the role of SIL.

## Materials and methods

### Study design and participants

This study was designed by the NHC Key Laboratory of AIDS Prevention and Treatment at the First Hospital of China Medical University and was approved by the Medical Ethics Committee of the First Hospital of China Medical University ([2023]No.41). All participants provided written informed consent. The participants were recruited from April 2023 to September 2024, including a baseline screening period followed by a 12-month follow-up including immune adjunctive treatment phase. All patients were administered by SIL (Silybin capsules, Tianjin Tasly Pharmaceutical Co. Ltd. orally at a dosage of 140 mg daily) for a duration of three months due to liver damage. The inclusion criteria were as follows: (1) participants aged 18 y or older; (2) a diagnosis of HIV infection in accordance with the Chinese HIV/AIDS treatment and diagnosis guidelines; (3) receipt of standard antiviral treatment for a minimum of 2 y, with a viral load of less than 20 copies/ml and CD4^+^ T-cell counts below 500/μL; (4) the presence of fatty liver disease or abnormal liver function (the indications for SIL); and (5) good adherence to SIL treatment. Prior to treatment, demographic information, including gender, ethnicity, age, weight, education level, occupation, and route of infection, as well as past medical history, smoking history, drug abuse history, hepatitis B markers, hepatitis C antibodies, B-ultrasound, chest X-ray, and electrocardiogram results, was collected. During baseline and follow-up, T-cell subset, routine blood tests, and blood biochemistry were tested. In order to evaluate the heterogeneity of SIL response, the participants would be grouped by the criteria as follows: compared with the baseline CD4^+^ T-cell counts, individuals whose CD4^+^ T-cell counts increased by more than 50 cells/µL and/or by more than 20%^1^ after SIL treatment were classified into the responsive group (SIL-R); individuals whose CD4^+^ T-cell counts remained unchanged or decreased following SIL treatment were defined as the non-responsive group (SIL-NR).

### Plasma collection

Fresh peripheral blood (20 ml) was collected in Ethylenediaminetetraacetic acid (EDTA) tubes, and plasma was isolated by centrifugation at 300 g for 10 min and then stored at −80 °C until utilization.

### Peripheral blood T lymphocyte subsets counting

10 µl CD4/CD8/CD3 (340499, BD Company) were added to the bottom of the “TRUE COUNT” tube. 50 µl of mixed EDTA anticoagulant peripheral blood was added to the bottom of the absolute counter tube and stained at room temperature (20 °C−25 °C) for 15 min in the dark. A total of 450 µl × FACS Lysing Solution (349202, BD Company) was added to the tube to avoid light for 15 min, and the sample was detected by flow cytometry.

### Immune phenotype of T lymphocyte detected by flow cytometry

Peripheral blood was collected from patients at baseline and three months after SIL treatment, and peripheral blood mononuclear cells (PBMC) were extracted using the density gradient centrifugation method. PBMC were stained with the flow cytometry antibodies listed in Supplementary Table 1. Acquisition was carried out on a FACScanto flow cytometer (BD Biosciences). The analysis was performed with FlowJo version 10 (Tree Star Inc., Ashland, OR, USA).

### Fecal sample collection

The fecal samples were collected at the initiation of SIL treatment. All fresh samples were stored in sterile containers and frozen at −80 °C until utilization.

### Fecal 16S rDNA sequencing

#### DNA extraction and 16S rDNA sequencing

DNA was extracted using CTAB from the fecal samples. The V3-V4 regions of 16S rDNA were amplified by the barcoded pair of 341F (5ʹ-CCTACGGGNGGCWGCAG-3ʹ) and 805 R (5ʹ-GACTACHVGGGTATCTAATCC-3ʹ). The PCR products were identified using 2% agarose gel electrophoresis and purified using AMPure XT beads (Beckman, USA). The amplicons were quantified by Qubit (Invitrogen, USA), and the size was assessed with an Agilent 2100 Bioanalyzer (Agilent, USA). The pooled libraries were sequenced on an Illumina NovaSeq PE250 platform.

#### Data processing and analysis

Based on sample barcodes, paired-end reads were allocated, and barcodes and primer sequences were removed. FLASH merged the reads. Quality filtering with fqtrim and Vsearch removed low-quality and chimeric sequences. DADA2 yielded an ASV feature table and sequences. The feature table was normalized with relative abundance. Alpha and beta diversities were determined after sequence normalization. Alpha diversity was assessed with five indices, including Chao1, observed species, Goods coverage, Shannon, and Simpson indices, which were calculated with QIIME2 to quantify within-sample species complexity, and rarefaction curve was plotted to represent the sequencing data and species richness. Compositional differences between samples (beta diversity) were assessed using principal component analysis (PCA) on centered log-ratio (CLR)-transformed relative abundance data by calculating Euclidean distances in this transformed space to summarize multivariate structure across samples. Compositional differences between samples (beta diversity) were evaluated by computing weighted-UniFrac distances and visualizing the dissimilarity matrix with principal coordinate analysis (PCoA). Sequence alignment was conducted with BLAST, and the feature sequences were annotated with the SILVA database (Release 138). The Mann‒Whitney *U*-test was used to define the differential bacterial genus, with *p*-value < 0.05. The linear discriminant analysis effect size (LDA effect size, LEfSe) was used to characterize the taxonomic biomarkers, with the LDA score >2 as the degree of difference in the relative abundance of bacteria between the two groups. PICRUSt2 (Phylogenetic Investigation of Communities by Reconstruction of Unobserved States) was used to predict the functional abundance based on the MetaCyc database. All the results were performed and visualized using the OmicStudio tools at https://www.omicstudio.cn/tool

### Fecal metabolomics detection

#### Metabolite extraction and identification by LC–MS

The fecal samples were thawed on ice, and the metabolites were extracted using 80% methanol. The metabolites were separated using an ACQUITY UPLC T3 column (100 mm*2.1 mm, 1.8 µm, Waters, USA) with a 3000 UPLC system (Thermo Fisher Scientific, Germany) and then detected using a Q-Exactive mass spectrometer in both ion modes. Precursor spectra and fragment spectra were collected at 70,000 and 17,500 resolutions, respectively.

#### Data processing and analysis

The MS data pretreatments were acquired using XCMS software. The raw data were converted into mzXML and processed using XCMS, CAMERA, and metaX packages with R software. The retention time and m/z data were combined to identify each icon. The metabolites are annotated using the online KEGG and HMDB databases by matching the molecular mass data (m/z), as well as an in-house fragment spectrum library of metabolites provided by LC Bio Technology Co., Ltd. (Hangzhou, China). T tests were conducted to detect differences in metabolite concentrations between groups. Supervised PLS-DA was conducted through metaX to discriminate the different variables between groups, and the VIP value was calculated. A VIP cutoff value of 1.0 was used to select important metabolites. The pathway enrichment analysis was performed based on the RaMP-DB database in MetaboAnalyst 6.0 online tool (https://www.metaboanalyst.ca//upload/EnrichUploadView.xhtml).

### Measurement of plasma inflammation related proteins

The Olink multiplex proximity extension assay (PEA) inflammation panel, including 92 inflammation-related proteins (IRPs), was employed to quantify the inflammation level in the plasma samples. The data generated by Olink are presented as normalized protein expression (NPX) values, and their distribution is comparable to that of the log 2-transformed protein concentrations. Both the samples that did not pass quality control and the data that were below the lower limit of detection were removed from further analysis. The pathway enrichment analysis was performed using DAVID database (https://david.ncifcrf.gov/home.jsp).

### Prediction model of silybin response

A random forest classifier, implemented using the RandomForest package in R 4.1.3, was employed to identify the gut bacteria and metabolites that best discriminate SIL responders from nonresponders. The variable importance was ranked by the mean decrease accuracy, and the ten most influential features were retained to conduct receiver operating characteristic (ROC) analysis for predicting the silybin response. To balance predictive power with clinical utility, both every single variables and multivariable combinations were evaluated by the corresponding area under the curve (AUC) in ROC analysis.

### Statistical analyses

Paired *t*-tests and chi-square tests were conducted to assess differences in demographic factors and immune markers between the pre-SIL and post-SIL groups. Spearman correlation analysis was used to establish the relationships between clinical characteristics, differentially abundant bacteria, metabolites, and IRPs. *p*-value < 0.05 was taken as the threshold for statistical significance; * represents *p* < 0.05; ** represents *p* < 0.01; *** represents *p* < 0.001; **** represents *p* < 0.0001. All the statistical analyses were performed using SPSS v25 and GraphPad Prism v8.0.1.

## Results

### SIL increased CD4^+^ T cells in immunological nonresponders but the effects had heterogeneity

**Figure 1.**
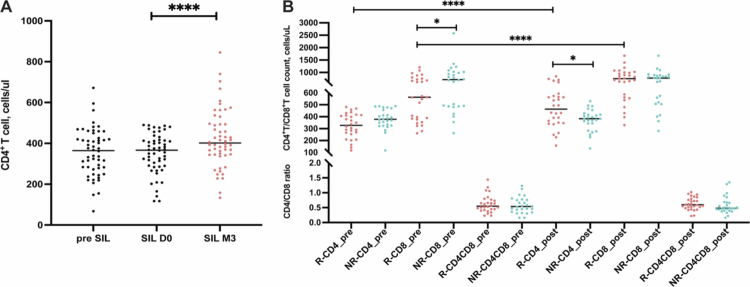
Clinical effects of SIL on HIV-positive immunological nonresponders. (A) The CD4^+^ T-cell counts at 6−12 months before SIL treatment (pre SIL), baseline of SIL treatment (SIL D0) and 3 months after SIL treatment (SIL M3) among 54 HIV-positive immunological nonresponders. (B) T-cell subsets, including CD4^+^ T-cell counts, CD8^+^ T-cell counts, and CD4/CD8 ratio before and after SIL treatment (pre and post) in SIL-R and SIL-NR. The red dots represent the SIL-R group, and the blue dots represent the SIL-NR group. *p < 0.05, and ****p < 0.0001.

To understand the response of SIL to immune reconstruction, 54 HIV-positive immunological nonresponders were orally administered SIL for 3 months, and T-cell subset counts and immune profiles at treatment initiation and three months were compared. At the end of the drug cutoff, the recovery of CD4^+^ T cells was observed, as CD4^+^ T-cell counts increased significantly after 3 months of SIL treatment (367 cells/µL vs. 401 cells/µL, *p* < 0.0001) (Figure 1A). Based on the recovery of CD4^+^ T-cell counts, the 54 patients were further classified as SIL-responders (SIL-Rs, *n* = 28) and SIL-nonresponders (SIL-NRs, *n* = 26). Notably, all individuals were men who had sex with men (MSM), and the demographic and clinical information, including age, BMI, HIV infection status, ART regimens, and durations, was similar in the SIL-R and SIR-NR groups. Although CD4^+^ T-cell counts before ART were statistically significant in the two groups, CD4^+^ T-cell counts were similar at the baseline of SIL treatment (Table 1). Except for CD4^+^ T-cell counts, the CD4/CD8 ratio at SIL treatment initiation was also not different, but CD8^+^ T-cell counts in the SIL-NR group were higher than those in the SIL-R group (Figure 1B). Interestingly, after SIL treatment, CD8^+^ T-cell counts also increased significantly in the SIL-R group but showed no change in the SIL-NR group (Figure 1B). Furthermore, immune phenotypes, including activation, senescence, and differentiation, were evaluated using flow cytometry at treatment initiation and after three months of SIL treatment. There were no differences in these T-cell phenotypes between the SIL-R and SIL-NR groups at treatment initiation (Figure S1A). However, the activation levels of CD4^+^ and CD8^+^ T cells were significantly reduced both in SIL-R and SIL-NR after SIL treatment (Figure S1B,C).

### The composition and functions of fecal bacteria varied in SIL-R and SIL-NR

**Figure 2.**
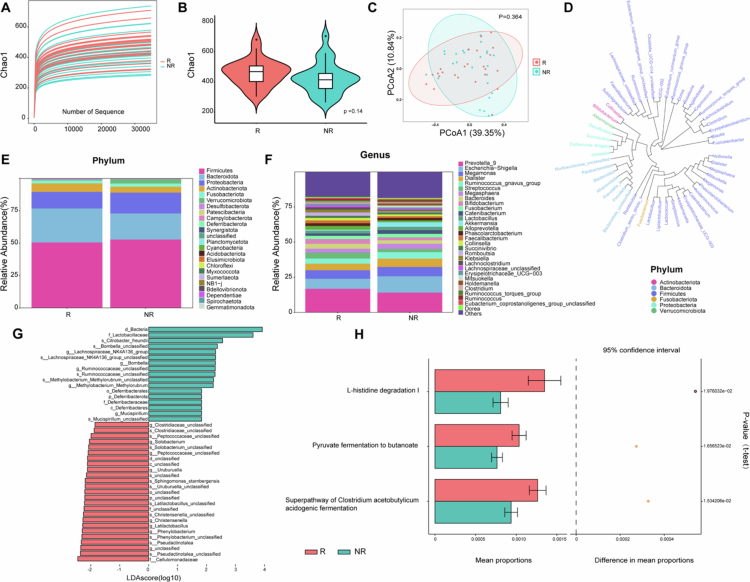
The composition and potential function of fecal bacteria at treatment initiation in SIL-R and SIL-NR analyzed by 16S rDNA sequencing data. (A) Rarefaction curve (Chao1 indices). (B) Alpha diversity (Chao1 indices). The error bars represent the distribution of the diversity scores. (C) Beta diversity using PCoA analysis. (D) The dependent relationships of phylum and genus constructed by phylogenetic tree. (E) The relative abundances of the bacterial communities at the phylum level. (F) The relative abundance of the bacterial communities at the genus level. (G) LEfse analysis of taxonomic biomarkers of the gut bacteria. The linear discriminant analysis score (LDA > 2) can be interpreted as the degree of difference in the relative abundance of ASVs between two groups. (H) Prediction of enrichment pathways based on the MetaCyc database using PICRUSt2 tool.

**Figure 3.**
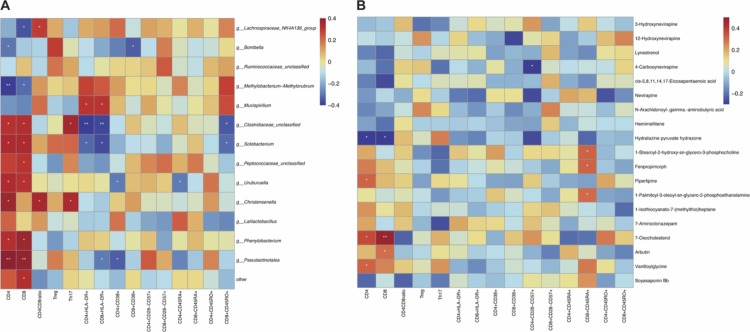
Correlation analysis (heatmap) of the changes in the clinical immune index with bacteria and metabolites. (A) Spearman's analysis between the relative abundance of 13 differential bacterial genera (Figure 2G) and the changes of clinical immune indices before and after SIL treatment. (B) Spearman's analysis between 19 differential metabolites (Figure 4A) and the changes of clinical immune indices. Red color represents positive correlations, and blue color represents negative correlations. *p < 0.05, **p < 0.01.

To investigate whether the gut (fecal) bacteria play a role in the different clinical effects of SIL on the immune reconstruction of INRs, the gut bacteria from all available samples (SIL-R = 24, SIL-NR = 25) at treatment initiation were analyzed using 16S rDNA sequencing, and the acquired data were sufficient for further analysis (Figure 2A). Although the alpha and beta diversities in the two groups were similar (Figure 2B,C), the taxonomic profiles differed (Figure 2E,F). At the phylum level, Firmicutes, Bacteroidota, Proteobacteria, Actinobacteriota, Fusobacteria, and Verrucomicrobiota were dominant in both groups, with a high abundance of Bacteroidota and Actinobacteriota in SIL-R and a high abundance of Proteobacteria and Verrucomicrobiota in SIL-NR. At the genus level, the top ten bacteria in the two groups were consistent, including *Prevotella*, *Escherichia–Shigella*, *Megamonas*, *Dialister*, *Ruminococcus*, *Streptococcus*, *Megasphaera*, *Bacteroides*, *Bifidobacterium*, and *Fusobacterium*. Compared to SIL-NR, the abundances of *Streptococcus*, *Bifidobacterium*, *Alloprevotella*, and *Rombotusia* were higher and Akkermansia was lower in SIL-R. To further explore the different bacteria associated with SIL-R versus SIL-NR, LEfSe analysis was performed using the LAD score > 2 as the threshold (Figure 2G). At the genus level, *Pseudactinotalea*, *Phenylobacterium*, *Latilactobacillus*, *Christensenella*, *Uruburuella*, *Peptococcaceae_unclassified*, *Solobacterium*, and *Clostridiaceae_unclassified* were significantly enriched in SIL-R, whereas SIL-NR exhibited higher abundances of *Lachnospiraceae_NK4A136_group*, *Bombella*, *Ruminococcaceae_unclassified*, *Methylobacterium-Methylorubrum*, and *Mucispirillum*. Furthermore, the differential bacterial genus identified by the Mann‒Whitney *U*-test were nearly consistent with the LEfSe results (Figure S2). The functional prediction using PICRUSt2 based on the MetaCyc database inferred that the enrichment pathways were the L-histidine degradation I pathway, the pyruvate fermentation to butanoate pathway, and the superpathway of Clostridium acetobutylicum acidogenic fermentation (Figure 2H). What is more, the correlation analysis between the differential bacteria and the immune phenotypes revealed that most of the certain bacteria enriched in the SIL-R group were related to the increase in CD4^+^ and CD8^+^ T cells and the reduction of T-cell activation, such as *Pseudactinotalea, Solobacterium*, and *Clostridiaceae_unclassified* (Figure 3).

### The combination of gut bacteria and metabolites at treatment initiation could distinguish SIL-R and SIL-NR accurately

**Figure 4.**
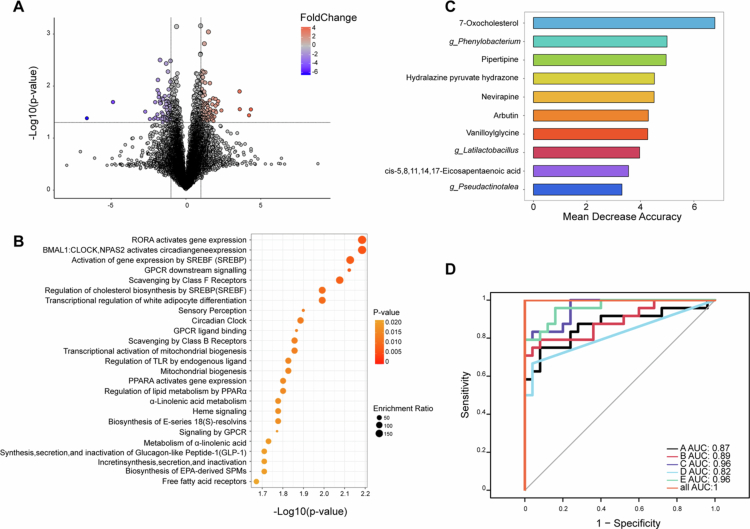
Differential fecal metabolites with related enrichment pathways, and the efficacy classifiers performed using bacteria and metabolites under random forest model. (A) The volcano map shows that 183 metabolites differentially expressed in the two groups were obtained by multivariate statistical analysis of PLS-DA (VIP > 1.0, *p* < 0.05, log 2 (FC) ≥ 1 or ≤ −1), highlighted with red and blue dots, respectively. (B) Enriched cell signaling pathways (top 25) of differential metabolites analyzed using the online pathway enrichment tool in MetaboAnalyst 6.0. (C) The random forest classifier was based on all differential bacteria and metabolites at treatment initiation, and the ten most influential variables are shown. (D) Area under the curve (AUC) of individual variables and different combinations. The combinations included *Phenylobacterium* with the most influential metabolite (7-oxocholesterol) marked by A; *Phenylobacterium* with the two most influential metabolites (7-oxocholesterol and Pipertipine) marked by B; *Phenylobacterium* with the four most influential metabolites marked by C; all three bacteria marked by D; and all seven metabolites marked by E.

To further determine the changes in gut bacteria-related metabolites, fecal metabolites from all available samples (SIL-R = 24, SIL-NR = 25) at treatment initiation were detected via untargeted metabolomics using LC-MS. In total, 183 differential ions between SIL-R and SIL-NR were identified from more than 20000 acquired ions, 19 of which were well annotated by KEGG, HMDB and an in-house fragment spectrum library of metabolites, including 10 upregulated and 9 downregulated metabolites in SIL-R (Figure 4A). The 10 upregulated metabolites were mainly belonged to lipids and lipid-like molecules (4/10) and organoheterocyclic compounds (2/10), whereas the 9 downregulated metabolites were mainly belonged to organic nitrogen compounds (4/9). Pathway enrichment analysis revealed that these annotated metabolites were enriched in RORA activates gene expression, BMAL1:CLOCK, NPAS2 activates circadian gene expression, activation of gene expression by SREBF (SREBP), regulation of cholesterol biosynthesis by SREBP (SREBF), circadian clock, mitochondrial biogenesis, PPARA activates gene expression and regulates lipid metabolism by PPARα (Figure 4B). Correlation analysis between the differential metabolites and the immune phenotypes revealed that most of the metabolites enriched in the SIL-R group were related to an increase in CD4^+^ and CD8^+^ T cells, especially 7-oxocholesterol (Figure 3B). Furthermore, random forest classifiers to identify the effect of SIL on the immune recovery of INR patients were developed by combining 13 differential bacteria and 19 differential metabolites at treatment initiation. The ten most influential variables, including 3 bacteria and 7 metabolites, were selected to perform ROC analysis (Figure 4C). The AUC was highest for the combination of ten variables, followed by the combination of seven metabolites (AUC = 0.96), *Phenylobacterium* combined with the four most influential metabolites (AUC = 0.96), *Phenylobacterium* combined with the two most influential metabolites (AUC = 0.89), *Phenylobacterium* combined with the most influential metabolite (AUC = 0.87), and all three bacteria (AUC = 0.82) (Figure 4D). Notably, the AUC can also reach to 0.78 when only using the most influential metabolite (7-oxocholesterol) (Figure S3). Allowing for the prediction power and model simplicity, the two models that combine all seven metabolites or one pairing *Phenylobacterium* with the four most influential metabolites serve as the most robust predictors of SIL response.

### The inflammation-related proteins in plasma related with gut metabolites and bacteria

**Figure 5.**
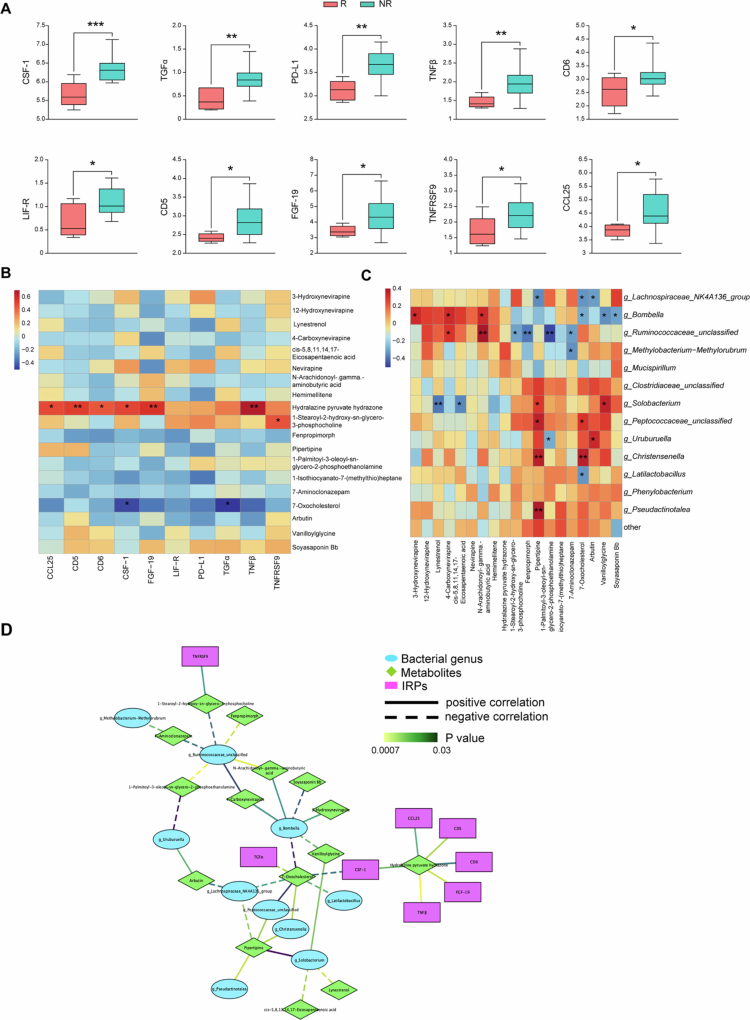
Differential plasma inflammation-related proteins (IRPs) between the two groups, and correlations among IRPs, gut metabolites and bacteria. (A) IRPs in SIL treatment initiation were detected and analyzed using Olink proteomics. The statistical significance between two groups was measured using a two-sample *t*-test after quality control. The results are represented by median normalized protein expression (NPX). Red and blue represent the SIL-R and SIL-NR groups, respectively. (B and C) Spearman's correlation analyses were conducted between 10 differential IRPs, 19 differential fecal metabolites, and 13 differential fecal bacteria. Red and blue represent positive and negative correlations, respectively. (D) The interactive network of differential bacteria, metabolites, and IRPs. *p < 0.05, **p < 0.01, and ***p < 0.001.

Allowing for the important roles of the gut bacteria in inflammation, plasma IRPs at treatment initiation were evaluated using Olink proteomics to analyze whether the inflammation situation of INRs can be modulated by gut bacteria or metabolites and further impact the effects of SIL. Among the 53 proteins with valid detections in 23 patients, 10 IRPs were significantly different between SIL-R and SIL-NR, all of which were downregulated within SIL-R (Figure 5A). KEGG enrichment analysis using DAVID showed that the most represented pathways were “Ras signaling pathway,” “MAPK signaling pathway” and “PI3K-Akt signaling pathway,” of which the related proteins were CSF-1, TGFα, and FGF-19, suggesting that these biological processes may impact SIL roles on immune recovery.

The correlation between IRPs and gut metabolites revealed that most IRPs were positively related to Hydralazine pyruvate hydrazone enriched within SIL-NR, and two IRPs (CSF-1 and TGFα) were negatively related to 7-oxocholesterol enriched within SIL-R (*p* < 0.05) (Figure 5B). Notably, 7-oxocholesterol was also positively related to *Christensenella* (*p *< 0.01) (Figure 5C), suggesting that *Christensenella* and 7-oxocholesterol may influence some immune modulation pathways by inhibiting the expression of CSF-1 and TGFα (Figure 5D).

## Discussion

Although ART effectively controls HIV-1 viremia, the immune response of some individuals cannot recover to normal levels. A large number of immune adjunct drugs have been used to increase CD4^+^ T cells and rectify the inflammation and immune activation of HIV-1 INRs; however, no candidate medicines have been tested to be fully effective. In this study, we first identified the effectiveness and heterogeneity of SIL on HIV immune recovery and then constructed an efficacy classification model based on the different gut bacteria and metabolites acquired at the baseline of SIL treatment. We also inferred that the interactive network of bacteria-metabolite-inflammation proteins may play key roles in promoting CD4^+^ T-cell recovery induced by SIL among INRs.

This study of 3-month SIL treatment for INRs revealed that SIL can increase CD4^+^ T cells and reduce T-cell activation. However, at the individual level, almost half of the INRs did not achieve immune recovery. Notably, in addition to the obvious increase in CD4^+^ T cells, CD8^+^ T cells were also significantly increased in the SIL-R group after SIL treatment (Figure 1B). Although there were no differences in the immune phenotypes of circulating T cells before and after adjunctive treatment between SIL-R and SIR-NR (Figure S1A–C), some IRPs of SIL-NR were higher than those of SIL-R (Figure 5A), of which CRF-1, TGF*α*, and FGF-19 were enriched in three cell signaling pathways. Interestingly, the “MAPK signaling pathway” and “PI3K-Akt signaling pathway” have been reported to be related to the anti-inflammatory and antitumor mechanisms of SIL.^10^^,^^25^ The “Ras signaling pathway” is located upstream of the “MAPK signaling pathway” and “PI3K-Akt signaling pathway” and can transmit signals by recruiting and activating downstream effectors such as Raf-1 and PI3K to regulate cell proliferation, differentiation, and death.^26^ Moreover, Wan et al. used the Olink multiplex proximity extension assay (PEA) inflammation panel to characterize inflammation levels in chronic HIV-1 patients with different clinical statuses and found that CSF-1 was negatively correlated with CD4^+^ T-cell counts and the frequency of native T cells, while it was positively correlated with the frequency of PD-1^+^ T cells.^5^ These results suggest that over-inflammatory activation of SIL-related pathways at treatment initiation may increase the threshold of the SIL-immune interaction and weaken the effectiveness of SIL, which has been observed in SIL-NR. Increasing the SIL dose or intervening early in these signaling pathways may improve the efficacy of SIL.

The gut bacteria perform various biological functions for health maintenance and disease development by regulating inflammatory molecules.^27^ An altered microbiota can induce a number of inflammatory disorders; on the other hand, bacteria-related metabolites, such as short-chain fatty acids (SCFAs), can exert anti-inflammatory and immunomodulatory effects.^28^ For the bacteria enriched in SIL-R, *Latilactobacillus* and *Christensenella* can produce acetate, propionate, and butyrate, and exert anti-inflammatory effect by inhibiting the NF-kB and MAPK signaling pathways; induce the expression of proinflammatory factors (e.g., IL-8), the production of reactive oxygen species, and the modulation of sugar, lipid, and energy metabolism.^29-31^ Furthermore, the high abundance of *Clostridiaceae* in SIL-R was consistent with the metabolic function predicted by PICRUSt2; that is, the superpathway of Clostridium acetobutylicum acidogenic fermentation predominated in SIL-R (Figure 2H). In addition, some intermediate metabolites from three different metabolic pathways (Figure 2H), such as pyruvate, acetyl-CoA, and fumarate, are located in the TCA cycle and play an important role in modulating inflammation and immunity except for energy supply and substance metabolism.^32^ In particular, fumarate is defined as an inhibitor of cell pyroptosis, and its derivative (dimethyl fumarate) serves as a candidate for treating inflammation and oxidative stress induced by viral infection.^33^^,^^34^ While SIL-R covers more beneficial bacteria, it is clear that they are unable to increase CD4^+^ T cells alone when no SIL is administered, suggesting that the gut bacteria in SIL-R provide a more suitable response environment for SIL to take effect.

The differences in fecal metabolites between SIL-R and SIL-NR would further explain how the gut bacteria improve SIL efficacy. These metabolites are enriched in some signaling pathways that affect the expression of circadian rhythm-related genes (Figure 4B). Retinoic acid receptor-related orphan receptors (RORs) can enter directly into the cell nucleus and regulate the expression of circadian rhythm-related genes, such as BMAL1 and CLOCK NSPA2, which drive various physiological processes, including metabolism and the immune system, and further influence health and diseases.^35^^,^^36^ The transcription factors (SREBF/P and PPARα) in the enriched pathways regulate lipid biosynthesis and metabolism and drive the lipid circadian rhythm in the liver,^37^^,^^38^ and they are also the molecular targets of SIL.^10^ Additionally, SIL is a lipid-soluble drug, and its biological activity may be affected by lipid biosynthesis and degradation induced by these various bacteria and metabolites. The circadian rhythm regulation of lipid metabolism induced by differential metabolites (Figure 4B) is also expected to increase the bioavailability and efficacy of SIL. Correlation analyses indicated that the metabolite 7-oxocholesterol was positively related to beneficial genera (*Christensenella*), CD4^+^ T cells and CD8^+^ T cells and negatively related to two IRPs (CSF-1 and TGF*α*) (Figures 3B and 5B,C). 7-Oxocholesterol is generally believed to be a toxic oxysterol from cholesterol, which has been researched in the initiation and development of cardiovascular and arterial diseases.^39^ However, one clinical research in breast cancer revealed that the selective changes of oxysterol in plasma were observed, such as an increase in 7-Oxocholesterol after tumor removal.^40^ 7-Oxocholesterol, an agonist of RORs, exerts an antiproliferative effect on tumor cell growth by modulating the circadian rhythm of lipid biosynthesis and degradation.^41^^,^^42^ Similarly, 7-oxocholesterol may also regulate inflammation, which is related to the low levels of some IRPs in SIL-R, especially CSF-1 and TGFα (Figure 5B). Taken together, the microbiota in SIL-R induces a low inflammation situation directly by related metabolites or indirectly by regulating circadian rhythm and lipid metabolism, which may be more suitable for the SIL pharmacological process.

Owing to the heterogeneity of SIL efficacy, timely classification and intervention are more important for promoting SIL-induced CD4^+^ T-cell recovery. The ten most influential gut-related variables were screened using Random Forest model to distinguish SIL-R and SIL-NR, including seven metabolites and three bacteria (Figure 4C). Obviously, the classification efficacy using the seven-metabolite combination is higher than the three-bacteria combination (0.96 vs. 0.82), indicating that metabolites have a higher potential for predicting SIL efficacy (Figure 4D). The pair of *Phenylobacterium* plus the four most influential metabolites achieved equal predictive efficacy compared with all metabolites (Figure 4D). The integration of bacteria and metabolites could further improve diagnostic accuracy, with an AUC value up from 0.78 to 0.87, outperforming any individual marker (Figure 4D, Figure S3). These noninvasive biomarkers offer promising potential for the clinical use of SIL in correcting the T-cell dysregulation of INRs.

This study has several limitations. First, the functional prediction of the gut bacteria was based on 16S rDNA data, which may not be accurate compared with metagenomic sequencing. Second, IRPs were analyzed in less than half of the samples, which could bias the differences. Finally, the mechanisms by which the gut bacteria and metabolites at treatment initiation affect the heterogeneity of SIL efficacy in CD4^+^ T-cell recovery require further analysis.

In conclusion, we found that the gut microbiota and related metabolites significantly influence the response of SIL treatment among INRs, highlighting the potential of microbiome-based interventions in improving immune reconstitution induced by adjunctive therapies in this population. We propose that the favorable bacteria and metabolites within some INRs promote CD4^+^ T-cell recovery when SIL is used as the adjunctive drug, which may be mediated by the lower level of inflammation activation situation of SIL signaling pathways (e.g. Ras, MAPK, and PI3k-Akt). These findings shed light on a novel therapeutic approach for addressing immune dysfunction in HIV-positive INRs and have important implications for personalized medical strategies in the management of HIV infection.

**Table 1.** Clinical characteristics of enrolled 54 HIV positive INR participants in this study.

Demographics (mean ± SD)	*R* (*n* = 28)	NR (*n* = 26)	*p*
Age (years)	43.35 ± 10.42	48.15 ± 13.89	0.19
BMI	22.52 ± 2.85	22.50 ± 3.05	0.72
Liver function (U/L) (mean ± SD)			
ALT	66.27 ± 44.88	51.65 ± 42.68	0.21
AST	44 ± 22.42	40.54 ± 37.42	0.59
GGT	97.85 ± 62.38	129.92 ± 135.45	0.33
TBIL	10.75 ± 6.65	10.93 ± 6.25	0.76
DBIL	3.58 ± 2.43	3.58 ± 2.74	0.62
ART scheme classification (%)			
2NRTIs + INSTI	10 (35.71)	10 (38.46)	0.84
2NRTIs + NNRTI	16 (51.14)	14 (53.85)	0.81
2NRTIs + PI	2 (7.14)	2 (7.69)	1.00
ART durations (years, mean ± SD)	6.77 ± 2.64	7.58 ± 3.66	0.22
Time from HIV diagnosis to SIL initiation (years, mean ± SD)	7.92 ± 3.07	8.08 ± 3.84	0.63
CD4 before enrollment			
CD4 cell counts/uL before ART	216.64 ± 187.50	120.19 ± 108.81	0.03
CD4% before ART	15.54 ± 9.20	17.23 ± 41.03	0.83
CD4 cell counts/uL before received SIL	351.86 ± 143.88	369.50 ± 115.15	0.62
CD4% before received SIL	21.89 ± 6.40	22.65 ± 7.04	0.68

The independent sample *t*-test was employed to compare the difference between SIL-R and SIL-NR for normal continuous variables. The row-multiply-list chi-square test was used to determine the difference in the number of cases between groups. Statistical significance is set at *p* < 0.05 (two-tailed).

## Supplementary Material

Supplementary material Table S1

## Data Availability

The data that support the findings of this study is deposited in National Microbiology Data Center (NMDC) with accession number NMDC10019381 [https://nmdc.cn/resource/genomics/project/detail/NMDC10019381].
