# Supplementary material for: Gut microbiome-induced metabolites promote the role of Silybin as adjunctive drug in HIV-positive immunological nonresponders
Source: Gut Microbes Rep. 2025 Oct 23;2(1):2569789. doi: 10.1080/29933935.2025.2569789 (PMC12940151; doi:10.1080/29933935.2025.2569789)
Supplement: Supplementary material — Table S1 [file KGMR_A_2569789_SM2822.docx]

Table S1. The key resources used in this study

| Flow Cytometry Antibodies | SOURCE | IDENTIFIER |
| --- | --- | --- |
| CD3 FITC / CD8 PE / CD45 PerCP / CD4 APC | BD Biosciences | Cat#340499 |
| PerCP CD3 | BD Biosciences | Cat#552851;Clone:SP34-2 |
| FITC CD4 | BD Biosciences | Cat#566911;Clone:SK3 |
| PE CD38 | BD Biosciences | Cat#555460;Clone:HIT2 |
| PE-Cy™7 CD8 | BD Biosciences | Cat#557750;Clone:RPA-T8 |
| APC CD25 | BD Biosciences | Cat#555434;Clone:M-A251 |
| PE CD127 | BD Biosciences | Cat#557938;Clone:HIL-7R-M21 |
| PE CD194 | BD Biosciences | Cat#551120;Clone:1G1 |
| APC CD196 | BD Biosciences | Cat#560619;Clone:11A9 |
| BV421 CD183 | BD Biosciences | Cat#562558;Clone:1C6/CXCR3 |
| PE-Cy™7 CD45RA | BD Biosciences | Cat#560675;Clone:HI100 |
| Pacific Blue™ CD4 | BD Biosciences | Cat#558116;Clone:RPA-T4 |
| APC CD45RO | BD Biosciences | Cat#559865;Clone:UCHL1 |
| FITC CD57 | BD Biosciences | Cat#555619;Clone:NK-1 |
| PE CD28 | BD Biosciences | Cat#555729;Clone:CD28.2 |
| APC-Cy7 CD8 | BD Biosciences | Cat#557760;Clone:RPA-T8 |
| APC HLA-DR | BD Biosciences | Cat#5599868;Clone:TU36 |
| Fixable Viability Stain 700 | BD Biosciences | Cat#564997 |
